# Progress in Surgery

**Published:** 1901-09-07

**Authors:** 


### Progress in Surgery.

(*Continued from page* 365.)

**Extraperitoneal Rupture of the Bladder.**—A. T. Bristow[3] reported a rare case of this injury to the Brooklyn Surgical Society. The patient, a woman of 24, fell down stairs and struck her abdomen against the corner of an ice-chest. She came to hospital 48 hours after. She said she had passed no urine since the accident, and had a constant desire to micturate. There was moderate tenderness and fulness of the abdomen, but no signs of peritonitis. Two ounces of urine were drawn off with a catheter. Then six ounces of salt solution were injected and the same amount recovered. For this reason laparotomy was put off. On the following morning two ounces of urine were again obtained. The abdomen was opened and sponge passed into each flank. No urine was found. The peritoneum was therefore closed and an investigation made of the anterior bladder wall. A collection of ammoniacal urine was found which had dissected its way on each side between the pelvic fascia and the peritoneum. For better exploration the recti were partly divided transversely at their pubic attachments. Excellent exposure was then obtained. Air was pumped into the bladder through a catheter, and by the bubbling it produced Bristow was enabled to find the rent in the bladder wall. The rent was about three inches long, and extended almost down to the bladder neck. It was sutured with great difficulty. Free drainage from the areas of extravasation was provided, and a catheter used to drain the bladder. Once there was leakage after the catheter had been left out. An excellent recovery was made. Commenting upon the case Bristow alluded to the extreme rarity with which extra-peritoneal rupture of the bladder was produced without fracture of the pelvis. In Mitchell's collection of 90 cases of extra-peritoneal rupture of this viscus, reported in 1898, there was fracture of the pelvis noted in all but three. It is rare for this form of rupture to be produced in any other way than by broken fragments of the pelvis being driven inward. Bristow had delayed operating for twelve hours in his case, because all the solution he put into the bladder had been returned. He considered that an excellent evidence of rupture of the bladder was the withdrawal of a constant small quantity of urine. It was explained by the bladder filling up to the point of rupture, and then overflow taking place into the surrounding tissues or peritoneum. A number of these cases had been treated by continuous immersion in a bath. Bristow secured perfect drainage from the bladder in his case through Dawbarn's apparatus.

**Enlargement of the Prostate.**—P. J. Freyer[4] has published two clinical lectures on this subject. In examining the gland from the rectum it is recommended that the patient should be placed on the couch on his knees with the thighs flexed and the buttocks rendered prominent. This position renders the prostate more prominent than the recumbent one, and the finger can be introduced further. For maintaining the sterility of catheters the use of trioxymethylene is mentioned favourably. This substance is a white powder which gives off formol as vapour. Catheters can, when dry, be placed in an air-tight box containing the powder enclosed in lint, and they will be quite aseptic in 24 hours. They must be rinsed in boric lotion before use. Carbolic oil is not recommended as a lubricant. Guyon's pomade, composed of equal parts of glycerine, powdered soap, and water, with 1 per cent. of phenol or naphthol, is spoken of as a clean and efficient lubricant. The catheters should be well washed with soap and warm water after use. Speaking of catheter fever Freyer says that it certainly occurs under the strictest antiseptic precautions, and with the utmost skill in passing the catheter. As bladder washes for cystitis the following are favourably mentioned: boric acid solution of 1 per cent. strength, or made by adding a teaspoonful of boroglycerine to four ounces of water; permanganate of potash, beginning with a 1 in 5,000 strength and working up to 1 in 1,000, and perchloride of mercury 1 in 10,000. But nitrate of silver solution is the sheet anchor. It is used very weak, 1 in 4,000 at first, and the strength gradually increased to 1 in 750. The bladder will not often tolerate a stronger solution than that. Resorcin, in 3 to 5 per cent. strength is also praised. In washing out not more than two or three ounces should be injected. For great pain and scalding at the bladder neck nothing is said to equal the treatment by "instillation" daily of strong nitrate of silver solution, 1 to 3 per cent., with the aid of Guyon's syringe passed just into the membranous urethra. Freyer says he has frequently known a pre-prostatic pouch—one lying in front of the middle lobe and bounded on each side by the lateral lobes—to be mistaken for the true bladder cavity. A case of the kind is mentioned in which the medical attendant could only draw off about half an ounce of urine each time he passed the catheter, as he thought, into the bladder. When the catheter was passed into the true bladder cavity three pints of urine escaped. Stones sometimes form in this pouch, and have even been removed by litholopaxy in it, the cavity being large enough to permit the working of a child's lithotrite. Freyer has met with two cases in which there was the complication of a calculus lying in a saccule of the bladder projecting out from the base of the bladder and leading to intense agony with defæcation. The mortality of the operation of castration for enlarged prostate is estimated at 20 per cent. This is probably owing to the advanced age of the patients, and their broken-down constitutions. In a considerable proportion of cases the operation has failed to induce atrophy of the prostate, probably cases in which the enlargement was fibroid rather than adenomatous in character. Freyer has never seen an instance where the power of voluntary micturition was permanently restored after this operation, or castration, or vasectomy, if the patient had previously been entirely dependent on his catheter. Vasectomy will prevent the recurrent epididymitis which is liable to affect prostatic patients. H. V. Critchley Hinder,[5] discussing prostatic hypertrophy, mentions two cases in which he found the disease present at the ages respectively of 41 and 43 years of age. Also he has found a blockage sufficient to give twenty ounces of residual urine due to a prostate which appeared hardly at all enlarged as felt from the rectum, and on the other hand he has known a prostate the size of a goose's egg give rise to no symptoms. Everything depends on the size and relationship of the obstruction to the internal orifice of the urethra. In one case of Hinder's the prostate being very large and the urethral orifice lifted up very high, very little urine could be drawn off unless the patient stooped forward and as it were emptied his bladder into the catheter. In another case mentioned, a man assisted himself to micturate by passing a small broom-handle into his rectum. Two or three of Hinder's patients have completely recovered from impotence after prostatectomy. Boracic acid administered by the mouth is said to be liable to produce albuminuria and mental peculiarities if given for longer periods than a fortnight. The author considers that perineal prostatectomy has a very limited field, and cannot relieve the cases where pedunculated growths exist in the bladder. The operation recommended is that of removing the obstructing portion of the prostate, not its whole bulk, by the suprapubic route, and passing a metal tube through the perineum for drainage.

**The Bottini Operation for Prostatic Obstruction.**—This subject was discussed at the February meeting of the New York Medical Association. L. Bolton Bangs[6] remarked that any surgical attack on the prostate gland was a serious matter. The more radical the operation the greater was the risk. The Bottini operation was a serious one. It was also not so simple as it would appear, and was an operation of skill, each detail requiring unrelaxed attention. The best guarantee against serious secondary hæmorrhage was slowness in operating. The following symptoms were liable to follow the operation. Often, for a day or two, there was great frequency of micturition and scalding pain with it. Some blood was often passed for a day or two. After one to three weeks some secondary hæmorrhage was to be expected. From the tenth to the fourteenth day burned tissues, generally in the form of minute black or brown particles, appeared in the urine. Fever followed the operation in 10 cases out of 34. Occasionally small sloughs which have separated can only be ejected from the urethra with difficulty. Epididymitis sometimes occurred. Three weeks were usually required for convalescence. Certain accidents and complications might follow the operation. In two of Bangs' cases incontinence of urine followed, and in one of these there was apparently occlusion of the seminal ducts. As to results, 60 per cent. of those operated upon by the author have discontinued the use of the catheter, having no residual urine or only a drachm or two. Twenty per cent. received no benefit. The operation was repeated on three patients, but only one of these derived benefit. Bangs has had two deaths directly attributable to the operation, and one to acute sepsis. Two other deaths occurred about three weeks after the operation, one from cardiac attacks, the other from uræmia after a debauch. As to the permanency of the results, time only will tell. Bangs cannot explain his 20 per cent. of failures. Willy Meyer said that he had operated upon 39 cases. In about 75 per cent. of these gonorrhœa had been present, which was interesting as it had been recently declared that there was reason to believe that gonorrhœa was largely responsible for the occurrence of prostatic enlargement.

[3] Brooklyn Med. Jour., January 1901. [4] Lancet, January 1901. [5] Australasian Med. Gazette, January 1901. [6] Med. Rec., March 1901.

